# Analysis of patient access to orphan drugs in Turkey

**DOI:** 10.1186/s13023-021-01718-3

**Published:** 2021-02-06

**Authors:** Güvenç Koçkaya, Sibel Atalay, Gülpembe Oğuzhan, Mustafa Kurnaz, Selin Ökçün, Çiğdem Sar Gedik, Mete Şaylan, Nazlı Şencan

**Affiliations:** 1 Econix Research, Analysis and Consulting Inc., İstanbul, Turkey; 2 Gen Pharmaceuticals and Health Products Inc., İstanbul, Turkey; 3 Ondokuz Mayıs University, Samsun, Turkey; 4 Bayer, Turkey; 5 Acıbadem University, İstanbul, Turkey

**Keywords:** Rare disease drug, Orphan drug, Orphan drug market

## Abstract

**Background:**

Rare diseases are life-threatening, serious, and chronic conditions that require complex care and have a low prevalence. An estimated one in 15 people worldwide are affected by rare diseases. This study aims to analyze the accessibility, reimbursement status, licensed status, and Anatomical Therapeutic Chemical (ATC) codes of drugs that the European Medicines Agency (EMA) in Turkey considers to be “orphan” pharmaceuticals.

**Methods:**

The drugs included in this analysis were obtained from the list of orphan drugs published by the EMA. Orphan drugs’ accessibility and licensing status in Turkey were obtained from the *Health Implementation Communiqué* published by the Social Security Institution (SGK) and the *List of Abroad Active Substance* and *List of Licensed Products* published by the Turkey Pharmaceuticals and Medical Devices Agency (TİTCK). Descriptive analysis was applied to determine the accessibility status of orphan drugs identified by the EMA in Turkey.

**Results:**

Based on the EMA, 105 pharmaceuticals were approved with “orphan drug” status except for drugs that have lost orphan drug status, decommissioned in the European Union and withdrawn from the European Community Register by January 2020. Of the 105 rare drugs on the EMA list, 34 were inaccessible in Turkey. Of the 71 available drugs, 23 (32%) were licensed and 48 (68%) were unlicensed in Turkey. 17 (74%) of licensed products and 17 (35%) of unlicensed products were covered by reimbursement. When orphan drugs’ ATC codes were examined, the most common ATC group was found to be “L—Antineoplastic and Immunomodulatory” agents.

**Conclusion:**

An orphan drug incentive policy is very important to ensure early access to the drugs used to treat rare diseases. Considering the capacity and prices for orphan drugs in Turkey, it can be said that many patients with rare diseases have difficulty in their treatment. It is obvious that such a policy must prepare for the regulation of orphan drugs in Turkey.

## Introduction

Rare diseases may seen rare in society, however they are one of major health problems as life-threatening, serious [1], chronic [2], complex [3], and demanding [4] conditions. Rare diseases are usually genetic in origin—such as cancer, autoimmune diseases, and degenerative and proliferative diseases—except for rare diseases originating from infection and infestation [1].

Although the definition of “rare disease” varies, diseases that affect fewer than 200,000 people in the United States (US), 50,000 people in Japan, and 2,000 people in Australia are considered “rare” [2, 5]. The World Health Organization (WHO) and EMA, considers a disease to be “rare” if it affects fewer than five people per 10,000 [2]. In general, rare diseases range in prevalence from one to eight people per 10,000 [5].

World epidemiological data are constantly changing. Although a new rare disease is discovered almost every week, its status may change from being considered a rare disease to being described as a common disease [1].

On the one hand, because rare diseases occur in unusual forms and often entail comorbidities, they are difficult to diagnose.

### Orphan drugs

An “orphan drug” is a drug especially developed to treat a rare medical condition. The high costs of drug development and pharmaceutical companies’ reluctance to develop drugs for very small patient populations make the public sector’s participation in the orphan drug market critical [6].

In 1581 Rembert Dodoens wrote “Medicinalium observationum exempla rara, recognita et aucta” a Latin book about the diagnosis and treatment of disorders with a low prevalence. This book, written by Rembert Dodoens, reveals the fact that studies of rare diseases date back 500 years [7]. The *Orphan Drug Act* was adopted in 1983 in the US to facilitate the research, development, and commercialization of drugs to treat rare diseases that are largely ignored [8]. Orphan drug legislation and policy have succeeded in promoting the progression of treatments for rare diseases. Since these policies’ implementation, more orphan drugs have been licensed in the US [9, 10].

### The orphan drug market

**Fig. 1:**
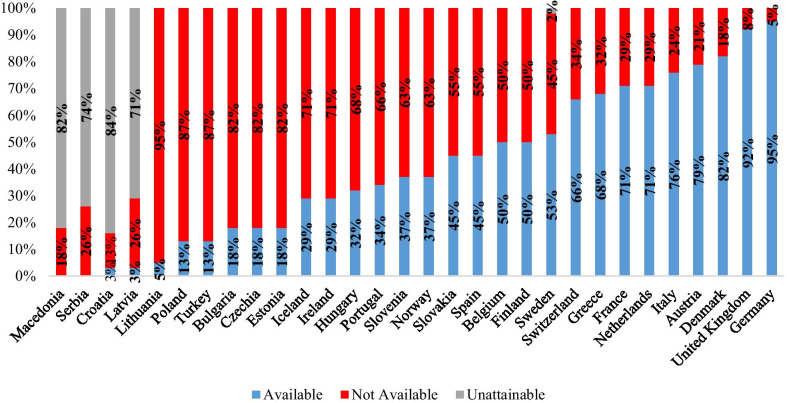
Availability rate measured by the number of drugs available since 2018 [13]

Marketing authorization for a drug does not mean that the drug is available in all countries within the European Union [11]. Marketing authorities determine commercialization status in each country. The necessary procedures are then applied to determine a drug’s reimbursement conditions and price [12]. In most countries, access to orphan drugs is only possible if it is included on the country’s reimbursement list. Figure 1 compares the number of orphan drugs on the market that are unavailable in European countries [13].

### Rare diseases and orphan drugs in Turkey

Rare diseases occur frequently in Turkey due to disease burden, consanguineous marriages, and different ethnic structures [11]. In Turkey, the Ministry of Health on the Pricing of Medicinal Products for Human Communiqué (2007) defined “orphan drugs” as drugs for the treatment of a disease affecting a population of less than 100,000 [14].

In Turkey, the TİTCK is following orphan drug operations in three ways. A drug:

1. Can be licensed and available for purchase on the market;
2. Can be currently not licensed in Turkey but available by prescription if approved in the US or the EU, efficacy and safety grounds are provided, and a clinical trial protocol is available;
3. Can be available for patients approved under the compassionate use program or with early access to a humanitarian medication to be clinically administered [8, 15].

In Turkey, in accordance with the *Regulation on Medicinal Products for Human Use Permit*, the Ministry of Health completes a preliminary examination within 210 days of the date an application is submitted. If the drug complies with legal regulations, its application is finalized [16].

Orphan drugs that are not approved for use for a specific indication and not licensed by TİTCK are delivered to the needy patient through an early access procedure in Turkey. This procedure allows physicians to prescribe off-label medications. Physicians wishing to prescribe an off-label medication must apply to TİTCK for named patient approval. TİTCK evaluates each application on the basis of published scientific evidence and recommendations of scientific committees. When TİTCK approves the use of an off-label drug, the import of the drug is provided by the Turkish Pharmacists Association [8].

This study aimed to analyze the accessibility, reimbursement status, licensed status, and Anatomical Therapeutic Chemical (ATC) codes of drugs considered “orphan” by the EMA in Turkey. In addition, the average price was calculated according to the number of boxes and prices of orphan drugs taken in Turkey based on ATC code by years. Accordingly, the study’s findings evaluated orphan drug policy and drugs’ access statuses.

## Method

### Data set

A general literature review was performed using Google Scholar, Google Books, the National Thesis Center, ProQuest, and Orphanet databases with the “rare disease” and “orphan drugs” keywords. In addition to the literature review, in order to perform the study’s analysis, a list of essential drugs was obtained from the official websites of the EMA, TİTCK, SGK, and the Association of Research-Based Pharmaceutical Companies. Turkey does not have an orphan drug list published by the Ministry of Health. For this reason, the drugs listed in the “List of Medicinal Products for Rare Diseases in Europe” published at www.orpha.net and www.orphadata.org were accepted as orphan drugs to be included in this analysis.

The official TİTCK and SGK websites were accessed for orphan drugs’ public cost data. Orphan drugs’ access and licensing statuses in Turkey were obtained from the *Health Implementation Communiqué* published by the SGK and the *List of Abroad Active Substance* and *List of Licensed Products* published by the TİTCK. Pharmaceutical costs were based on drugs’ sale prices. Costs provided by the SGK in Turkish lira and US dollars were converted into euros, according to the average exchange rate for 2019 published by the Ministry of Treasury and Finance.

### Data analysis

The necessary data were collected on license conditions, access to Turkey, ATC reimbursement restrictions, classification of diseases, and pricing for the quantities and budgets of medicines on the *Orphan Medicines List*.

Table 1 provides ATC codes and their descriptions. The ATC coding system provides the international coding and classification of all molecules that can be licensed and used as drugs. In this analysis, orphan drugs were evaluated on the basis of their ATC codes.

**Table 1:** ATC codes and descriptions

ATC codes	
L	Antineoplastic and ımmunomodulator agents
J	Systemic antiinfectives
A	Gastrointestinal canal and metabolism
B	Blood and blood making organs
M	Musculoskeletal system
N	Nervous system
V	Sundry
D	Drugs used in dermatology
R	Respiratory system
C	Cardiovascular system
S	Sense organs
H	Systemic hormone preparations (excluding sex hormones and ınsulin)

The compiled data were transferred to Microsoft Excel. A descriptive analysis was applied to the transferred data.

## Results

**Fig. 2:**
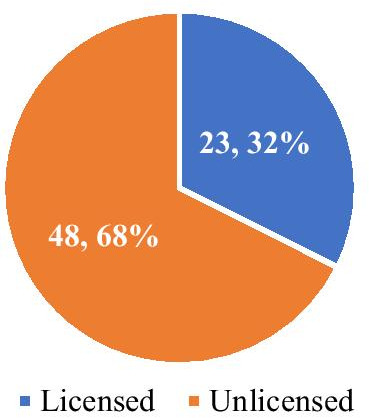
Licensed status of orphan drugs in Turkey

Based on the EMA, 105 pharmaceuticals were approved with “orphan drug” status except for molecules that have lost orphan drug status, decommissioned in the European Union and withdrawn from the European Community Register by January 2020. Access was unavailable in Turkey to 34 of the 105 molecules as orphan drugs on the EMA list (*List of Medicinal Products for Rare Diseases in Europe*). Therefore, our analysis evaluated 71 molecules which are orphan drugs. Of these 71 drugs, 23 (32%) were licensed in Turkey and 48 (68%) were unlicensed in Turkey (Fig. 2).

**Fig. 3:**
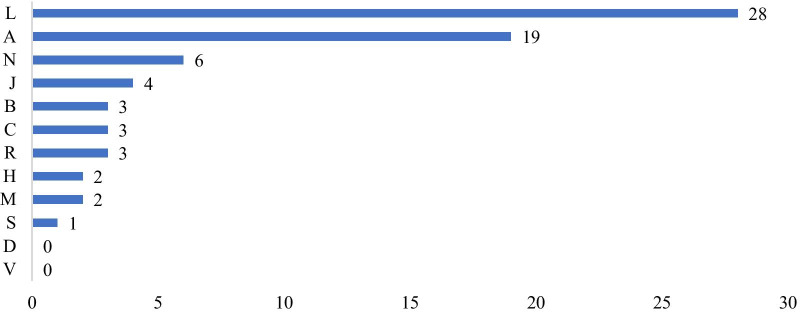
Distribution of all orphan drugs in Turkey by ATC code

Figure 3 shows the distribution of all orphan drugs based on their ATC codes. An examination of orphan drugs based on their ATC codes revealed that the most common ATC group was “L—Antineoplastic and Immunomodulatory” agents. This group usually relates to cancer and the immune system. The second group on the list after Code L was “A—Gastrointestinal Canal and Metabolism.” This group usually relates to gastrointestinal and metabolic diseases. There are 6 drugs with N ATC code in Turkey. This code group covers diseases of the nervous system, including neurological diseases. No orphan drugs with D or V ATC codes were available in Turkey.

**Fig. 4:**
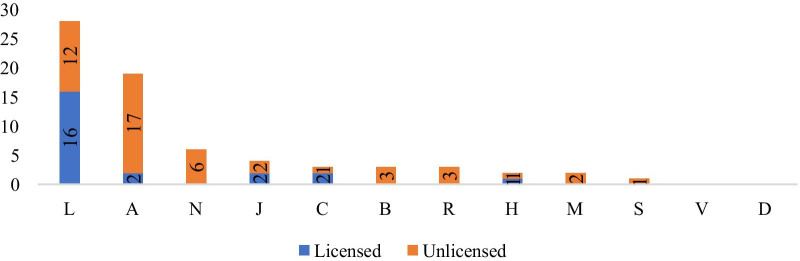
License status of orphan drugs that have access in Turkey based on ATC code

Figure 4 shows that 16 (70%) of 23 licensed products belonged to the L group. None of the B, D, M, N, R, S, or V ATC codes were licensed products. Of the 48 unlicensed products, 12 (25%) belonged to Group L and 17 (35%) belonged to Group A.

**Fig. 5:**
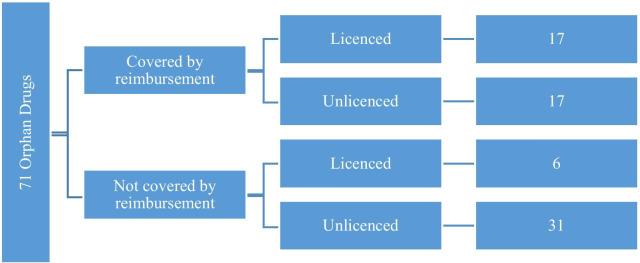
Reimbursement status of orphan drugs based on licenced status

17 (74%) of licensed products and 17 (35%) of unlicensed products were covered by reimbursement (Fig. 5). None of the products from abroad were covered by reimbursement in Turkey. Of the 71 drugs accessible in Turkey, 34 (48%) were covered by reimbursement. The rest were available for patients’ out-of-pocket payment .

**Fig. 6:**
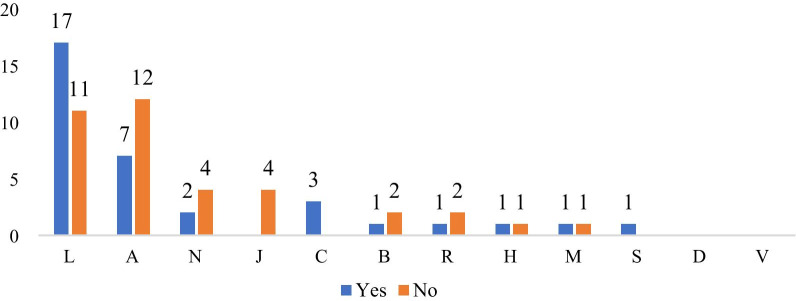
The situation of reimbursement of orphan drugs who have access in Turkey, based on the ATC code

An analysis on the basis of ATC codes revealed that most of the L-code drugs were included in the reimbursement scope (Fig. 6). The most important reason for this inclusion is that 34% of the products on the list were L-code. Although only 2 of the drugs in the A-code are licensed, it is seen that 12 of them are within the scope of reimbursement. None of the orphan drugs with J, V, or D ATC codes were covered by reimbursement.

Table 2 shows the average prices for orphan drugs on an ATC basis in 2016–2019. Drugs with access in Turkey were included in the analysis. It is analyzed that average prices increased over the years that investigated. This expected increase is due not only to an increase in drug prices but also to an increase in the number of drugs accessible in Turkey.

**Table 2:** Average price of orphan drugs based on ATC code by years in Turkey

ATC code	Price (€)			
	2016	2017	2018	2019
L	2,327.72	1,804.77	1,526.23	2,330.57
A	3,389.34	2,454.66	5,866.38	4,574.54
J	0	0	0	1,452.57
N	898.07	897.46	13,506.64	31,605.39
B	1,626.07	1,626.07	1,576.07	2,640.00
S	195.00	655.00	655.00	655.00
C	661.87	598.04	518.15	823.24
H	1,585.51	1,090.53	880.96	1,666.07
R	336.04	336.04	0	2,390.39
M	4,427.00	4,427.00	4,427.00	8,115.67
V	0	0	0	0
D	0	0	0	0
Average	1,802.38	1,554.21	3,111.47	3,907.32

## Discussion

Considering the population affected by rare diseases in each country, governments’ policies are important in the R&D and marketing processes for orphan drugs necessary for treatment [17]. The pharmaceutical industry has little interest in orphan medicinal products due to the low number of patients in normal market conditions. Therefore, the European Union provides incentives to pharmaceutical companies for research and development [18, 19].

In 2020, of the 105 orphan drugs on the EMA’s list, 34 are not accessible in Turkey. Of the 71 remaining items, 34 have reimbursement coverage in Turkey. When this rate is compared with other countries; It was observed that data on orphan drugs were not available for Macedonia and Serbia, and there was access to 5 orphan drugs in Lithuania and 13 in Poland. The countries with the highest number of access to orphan drugs are Germany, the United Kingdom, and Denmark [13].

When the reimbursement status of orphan drugs is examined; a study found that 88 of 165 orphan drugs in South Korea were covered by reimbursement as of 2019 [20]. There are 68 orphan drugs covered in England, 55 orphan drugs in Scotland and 47 orphan drugs in Wales [21]. A study found that 69% of 43 drugs that had access in Sweden were covered by reimbursement. On the other hand, 94% and 100% of all launched orphan drugs were reimbursed in Italy and France respectively. The reason for such high reimbursement rates in France and Italy is that reimbursement decisions in these countries are made based on literature reviews and cohort studies, not on cost-effectiveness analyzes. Countries that require a standard of evidence that includes a formal clinical and cost-effectiveness analysis generally have lower coverage than countries using alternative evidence standards [22].

The examination of orphan drugs’ ATC codes revealed the most common ATC group to be “L—Antineoplastic and Immunomodulating” agents. This group includes treatments for cancer and immune-system diseases. The second-most-common code was “A—Gastrointestinal Canal and Metabolism.” This group includes treatments for gastrointestinal and metabolic diseases. Similarly, it was observed that 42% of the products that received marketing approval for orphan drugs between 2008 and 2017 in the United States of Europe were in the field of oncology. This may be because products developed for rare cancers have a high profit potential, as they can also be applied to other types of rare/ non-rare cancers [23].

An examination of orphan drugs’ licensing status based on their ATC codes and access in Turkey showed that, of the 23 licensed products listed, 16 belonged to Group L, two to Group A, two to Group C, two to Group J, and one to Group H. Approximately 70% of licensed products were in Group L.

The examination of orphan drugs’ ATC codes based on their reimbursement status in Turkey showed that Group L (17 drugs) was included most often in reimbursement coverage. None of the four products in ATC Group J were available for reimbursement. Products with V and D ATC codes were not accessible in Turkey.

The most expensive product on the list of orphan drugs was the nusinersen active ingredient product. Nusinersen’s unit price was 90,000.00 euros. Pharmaceutical-market sales of this product in Turkey started in 2018, with only 21 boxes initially available. Nusinersen is a reimbursement product. Worldwide, after the US and Germany, Turkey has been granted third-place approval to use this drug for 593 patients diagnosed with spinal muscular atrophy (SMA) in the context of reimbursement coverage for drugs.

Because there is no definite data on the distribution of rare diseases in Turkey it is unknown what is needed most in need of care, but SMA disease that is the most up to agenda. The current situation in Turkey; the vast majority of people to be accessible to new gene therapy for the treatment of SMA and receiving reimbursements are requested from the political actors and decision makers. Some patients who cannot access the treatment they need in Turkey go abroad to be treated. But because this method requires a huge cost, very few people can go. In this reason, it is vital that pharmaceutical companies and decision makers make mutual agreements and meet patients' access to treatment as soon as possible. Due to the very high prices of orphan drugs, market access agreements between the pharmaceutical companies and the reimbursement agency will reduce the risk for both parties and provide patients with access to drugs.

An examination of the drugs’ list prices revealed their average price to be 1,802.38 euros in 2016, 1,554.21 euros in 2017, 3,111.47 euros in 2018, and 3,907.32 euros in 2019. The deviation in 2018 was due to Nusinersen’s unit price of 90,000.00 euros. The increase in average prices in 2019 was due to an increase in the number of drugs. A study found that the average annual Orphan Drug Price in the United States in 1998 was 7,136 USD and in 2017 was 186,758 USD [24]. It has been reported that in the US over the past 20 years, the market has shifted towards drugs that treat fewer people, such as those with rare diseases [23]. Considering the price increase between 1998 and 2020, it can be said that the USA concentrated on the orphan drug market.

In a study conducted in South Korea; the average spending on orphan drugs was found to be USD 27,275 in 2016, USD 41,682 in 2017 and USD 36,629 in 2019. The reason why average annual orphan drug spending in South Korea is so high may be due to the fact that 62,413 people have the rare disease as of 2019 [20].

The analysis-based ATC codes seemed to reveal that products in the L Group had the biggest price share and that, from 2018 to 2019, a significant increase occurred in the M, R, and J code groups.

Updating national policy for rare diseases and orphan drugs is critically important. For researchers, the incentives for conducting domestic clinical research on the diagnosis and treatment of rare diseases are an important part of such policy. Moreover, legal texts should be tightly regulated to allow patients faster access to treatment. Late diagnosis, delayed access to appropriate treatment centers, an inadequate number of drugs used in patients’ treatment, problems in supplying drugs, and high drug costs present difficulties for both patients and scientists in investigating these diseases. Rare diseases face many problems due to a lack of knowledge and experience, a lack of specialist physicians, and difficulties in patients’ treatment and follow-up. For these reasons, an effective unit of the Ministry of Health for rare diseases and orphan drugs should work actively to establish the necessary examinations, inspections, and relevant legislation. The cooperation and support of all responsible stakeholders—such as patients and patients’ relatives, physicians, specialists, political actors, and sector representatives—would play a major role in this initiative.

## Conclusions

An orphan drug incentive policy is very important to ensure early access to the drugs used to treat rare diseases. As a result of this study, it was found that not all orphan drugs are accessible in Turkey, and most of the drugs that are available are unlicensed products. Most of the unlicensed drugs are brought from abroad with an additional approval from the Rebuplic of Turkey Ministry of Health. This causes the patients' access to drugs to be slowed and delayed. Considering the capacity and prices for orphan drugs in Turkey, it can be said that many patients with rare diseases have difficulty in their treatment. It is obvious that such a policy must prepare for the regulation of orphan drugs in Turkey.

The literature includes no studies that have analyzed reimbursement statuses between 2016 and 2020 in terms of access to orphan drugs, annual use of orphan drugs, and the economic burden of orphan drugs in Turkey. Information on this topic is significantly lacking in the literature. Thus, this study constitutes an important source of information. The authorities and decision maker should take action for raising the awareness of the financial burden facing individuals with rare diseases and their families as well as their difficulties accessing necessary drugs.

## Data Availability

The datasets used and/or analysed during the current study are available from the corresponding author on reasonable request.

## Abbreviations

ATC: Anatomical Therapeutic Chemical
EMA: European Medicines Agency
HTA: Health Technology Assessment
SGK: Social Security Institution
SMA: Spinal Muscular atrophy
TİTCK: Turkey Pharmaceuticals and Medical Devices Agency
US: United States
WHO: World Health Organization
